# MARCH1-mediated ubiquitination of MHC II impacts the MHC I antigen presentation pathway

**DOI:** 10.1371/journal.pone.0200540

**Published:** 2018-07-12

**Authors:** Kayla R. Wilson, Haiyin Liu, Geraldine Healey, Vivian Vuong, Satoshi Ishido, Marco J. Herold, Jose A. Villadangos, Justine D. Mintern

**Affiliations:** 1 Department of Biochemistry and Molecular Biology, The University of Melbourne, Bio21 Molecular Science and Biotechnology Institute, Parkville, Victoria, Australia; 2 Department of Microbiology, Hyogo College of Medicine, 1–1 Mukogawa-cho, Nishinomiya, Japan; 3 Walter and Eliza Hall Institute, Parkville, Victoria, Australia; 4 Department of Medical Biology, The University of Melbourne, Parkville, Victoria, Australia; 5 Department of Microbiology and Immunology, Peter Doherty Institute for Infection and Immunity, The University of Melbourne, Parkville, Victoria, Australia; Universitat Hohenheim, GERMANY

## Abstract

Major histocompatibility complex class II (MHC II) expression and turn-over are regulated via its ubiquitination by the membrane associated RING-CH 1 (MARCH1) E3 ligase. Unexpectedly, we show that MHC II ubiquitination also impacts MHC I. Lack of MARCH1 in B cells and dendritic cells (DCs) resulted in a significant reduction in surface MHC I expression. This decrease was not directly caused by changes in MARCH1 ubiquitination of MHC I but indirectly by altered MHC II trafficking in the absence of its ubiquitination. Deletion of MHC II in *March1*^-/-^ cells restored normal MHC I surface expression and replacement of wild type MHC II by a variant that could not be ubiquitinated caused a reduction in MHC I expression. Furthermore, these cells displayed inefficient presentation of peptide and protein antigen via MHC I to CD8^+^ T cells. In summary, we describe an unexpected intersection between MHC I and MHC II such that the surface expression of both molecules are indirectly and directly regulated by MARCH1 ubiquitination, respectively.

## Introduction

Ubiquitination is a post-translational modification that acts as a sorting signal to deliver proteins to specific cellular destinations. For integral membrane proteins, ubiquitin attachment alters their traffic to and from the cell surface [1]. This occurs either by redirecting their route in the secretory pathway or via the regulation of endocytic trafficking. The ability of ubiquitin to control membrane protein localization and expression has significant consequences for cellular function. In the immune system, ubiquitination plays important regulatory roles [2].

Surface expression and half-life of major histocompatibility complex class II (MHC II), the molecule responsible for CD4^+^ T cell antigen presentation and initiation of adaptive immunity, is controlled via ubiquitination [3,4]. Ubiquitin is covalently attached to a single lysine in the MHC IIβ cytosolic tail [5–9]. The E3 ubiquitin ligase responsible is a member of the membrane-associated RING-CH (MARCH) E3 ubiquitin ligase family. MARCH1 ubiquitinates MHC II in haemopoietic cells, including dendritic cells (DCs) and B cells [8–12], while MARCH8 controls MHC II expression in thymic epithelium [13,14]. Ubiquitination of MHC II in both murine and human cells serves as a critical molecular switch that regulates its internalization and recycling to and from the cell surface [5,8,9,11,15]. Similar to MHC II, the surface expression of CD86, a major T cell co-stimulatory molecule, is regulated by MARCH1 ubiquitination [16,17]. The importance of MARCH mediated ubiquitination in the control of immuno-regulatory receptor trafficking is highlighted by the role of MARCH8 in the development of CD4^+^ T cells [13,14] and MARCH1 in the generation of regulatory T cells and DC function [12,18,19].

Here we describe a role for MARCH1 in the regulation of receptor expression that goes beyond ubiquitination *per se*. We describe a role for MARCH1 in the regulation of MHC I surface levels. MHC I is responsible for the presentation of antigen to CD8^+^ T cells and does so via pathways of direct (presentation of endogenous antigen) or cross-(presentation of exogenous antigen) presentation. We describe here that by ubiquitinating MHC II, MARCH1 promotes elevated MHC I surface expression that impacts both direct and cross-presentation by professional antigen presenting cells.

## Materials and methods

### Generation of mutant MuTu DC using CRISPR/Cas9

MuTu DC1940 cells were provided by Hans Acha-Orbea (University of Lausanne, Switzerland) [20] and cultured in Iscove’s Modified Dulbecco’s Medium (IMDM)-GlutaMax™ (Thermo Fisher) supplemented with 10% (v/v) FBS (University of Melbourne Media Preparation Unit), 100 μM β-mercaptoethanol (Thermo Fisher), 60 μg/mL penicillin and 100 μg/mL streptomycin at 37°C, 10% CO_2_. To generate a MuTu DC line stably expressing Cas9, the FUCas9Cherry lentivector (Addgene plasmid #70182), gifted by Marco Herold, was used [21]. Lentivirus was produced by standard HEK293T transfection before being spin cultured with MuTu DCs in the presence of polybrene (Sigma-Aldrich). mCherry^+^ cells were sorted using a BD Influx (BD Biosciences). sgRNAs were designed using the MIT CRISPR design software (http://crispr.mit.edu/) and cloned into the FgH1t_UTG vector (Addgene plasmid #70183), gifted by Marco Herold [21]. sgRNA sequences were as follows: hBIM exon 3: 5’ GCCCAAGAGTTGCGGCGTAT 3’; March1 exon 6: 5’ CTGAGCTCTTGATCCATTGG 3’. To generate MHC II knockout cells, MuTu DCs were transfected with 30μM synthetic crRNA:tracrRNA complex targeting H2-Ab1, using the AltR^TM^ CRISPR system according to manufacturer’s recommendations (IDT). The crRNA sequence used was 5’ CGACGTGGGCGAGCACCGCG 3’. The pMSCV-IRES-GFP II (pMIG II) was a gift from Dario Vignali (Addgene plasmid #52107) and was used for the expression of MHCII K225R [22]. MuTu DCs were transduced as previously described. CFP^+^ cells were sorted using a BD Influx (BD Biosciences) and sgRNA transcription with 1 μg/mL doxycycline (Sigma-Aldrich). Successful genomic cleavage of the target gene was confirmed using GeneArt Genomic Cleavage Detection Kit (Thermo Fisher). Expression of MHCII K225R in MHC II knock out MuTu DCs was achieved by transduction with pMIG II-MHCIIKR. MHC II K225R^+^ GFP^high^ cells were sorted to purity with a BD Influx (BD Biosciences).

### Mice

C57BL/6, *March1*^*-/-*^ [8], *I-Aα*^-/-^ [23], MHC II K225R^ki/ki^ [18] and OT-I mice [24] were bred and maintained in specific pathogen-free conditions at the Bio21 Molecular Science and Biotechnology Institute. Analysis was undertaken with mice aged 6–12 weeks. Mice were euthanized by carbon dioxide administration. Experiments were conducted in accordance with guidelines provided by National Health and Medical Research Council of Australia. Experimental procedures were approved by the Animal Ethics Committees at the University of Melbourne (Application 1513472).

### Flow cytometry

Cells were washed twice with EDTA-BSS with 2% (v/v) FBS and incubated with antibodies for 30 minutes at 4°C. Antibodies specific for MHC II (M5/114), MHC I H-2K (Y3), MHC I H-2K^b^ (AF6-88.5), MHC I H-2D^b^ (28-14-8), CD86 (GL-1), B220/CD45R (RA3-6B2), CD11c (N418), CD8 (53–6.7), CD8 (YTS169.4), CD3 (KT3-1.1) and TCRVα2 (B20.1) and conjugated to fluorochromes PE, FITC, APC, PE-Cy7, eFluor450 or BV421 were obtained from BioLegend. Cells were analyzed using a LSRFortessa apparatus (BD Biosciences) and data were analyzed with FlowJo software (Tree Star). Gating strategies for individual cell populations are shown in S1 Fig.

### Enrichment of primary B cells and dendritic cells

Sub-mandibular blood was collected and red blood cells were lysed. B cells were identified as B220^+^ cells. DCs were isolated as previously described [25]. Briefly, splenic tissue was digested with 1 mg/mL DNAse (Roche) and 7 mg/mL Collagenase Type III (Worthington Biochemical Corporation). Cell clusters were dissociated by treatment with 10 mM EDTA. Light density cells were isolated by centrifugation over a Nycodenz gradient (1.077 g/cm^3^; Nycomed Pharma) at 1,700 *g* and incubated with rat anti–mouse antibodies against CD3 (KT3-1.1), Thy1-1 (T24/31.7), CD45R (RA36B2), Ly6G (IA8) and erythrocyte (TER119; all produced at the Walter and Eliza Hall Institute antibody facility), followed by depletion with goat anti-rat IgG-coated magnetic beads (Bio-Rad). DCs were identified as CD11c^+^ cells. The purity of the dendritic cell isolations were 60–90% CD11c^+^. For antigen presentation assays, CD11c^+^ or CD11c^+^ CD8^+^ cells were sorted to > 90% purity using a FACS AriaFusion or Influx apparatus (BD Bioscience).

### Immunoprecipitation

1 x 10^7^ DCs were lysed in 1% (v/v) Triton X-100, 1 mM EDTA pH 8, 150 mM Tris-HCl pH 7.4, 10 mM N-ethylmaleimide (Thermo Fisher) and cOmplete™ protease inhibitor cocktail (Sigma-Aldrich). To reduce non-specific binding, the lysate was pre-cleared with protein G-sepharose beads (WEHI Antibody Facility) and normal rat and mouse serum (WEHI Antibody Facility) or protein G-sepharose alone. MHC II and MHC I molecules were precipitated with anti-MHC II (M5/114) and anti-MHC I (Y3) antibodies and protein-G sepharose beads. Proteins were eluted with non-reducing Laemmli sample buffer and analyzed by SDS-PAGE and western blotting. Western blots were probed using anti-ubiquitin antibody (P4D1, Santa Cruz Biotechnology), anti-MHC II antibody (JV2) or anti-MHC I antibody (Y3).

### Internalization assay

Fluorescence internalization probe (FIP)-azide (5′ Cy5- TCAGTTCAGGACCCTCGGCT-N3 3′) and quencher (Q; 5′-AGCCGAGGGTCCTGAACTGA-BHQ2 3′) were purchased from Integrated DNA Technologies. To determine MHC I and MHC II internalization FIP assays were performed as previously described [26]. Briefly, DCs were enriched and stained on ice with FIP-Cy5–conjugated anti-MHC II or anti-MHC I antibodies. FIP-labeled cells were incubated in complete RPMI 1640 at 37°C and 10% CO_2_ for 30 minutes. Cells were washed, phenotyped for surface markers (N418 for CD11c), and resuspended in media containing propidium iodide with or without 1 μM quencher (Q). The percentage of internalization was calculated with the equation below, where Q_n_ is the geometric mean fluorescent intensity (MFI) at time n after the addition of Q, Q_0_ is the MFI at time 0 after the addition of Q, and F_0_ is the MFI at time 0 without the addition of the Q.

$$\%internalisation=\frac{Q_{n-}Q_{0}}{F_{0-}Q_{0}}\times 100$$

Cells were analyzed using a BD LSRFortessa apparatus (BD Biosciences). Data were analyzed with FlowJo software (Tree Star).

### Imunofluorescence microscopy

1 x 10^5^ MuTu DCs were attached to coverslips treated with anti-MHC II antibody (10 μg/mL, N22) as described [27]. Cells were fixed with 4% paraformaldehyde/PBS, permeabilized with 0.3% Triton X-100 in PBS and blocked with 0.1% Triton X-100, 10% FCS in PBS. Cells were stained with rabbit anti-MHC I polyclonal antiserum (generated to the cytosolic tail of MHC I), donkey anti-rabbit Alexa Fluor 647 (Abcam) and 0.5 μg/ml DAPI (Thermo Fisher). Coverslips were mounted in SlowFade Diamond (Thermo Fisher) and analyzed on a Leica SP8 Confocal microscope (Biological Optical Microscopy Platform, University of Melbourne).

### Antigen presentation assay

Total lymph nodes were collected from OT-I mice [24] and single cell suspensions were generated. CD8^+^ T cells were isolated by incubating with rat anti–mouse antibodies against MHC II (M5/144), CD11b (M1/70), erythrocyte (TER119), F4/80 (F4/80), Ly6C/G (RB6-8C5) and CD4 (GK1.5, all produced at the WEHI Antibody Facility) followed by depletion with goat anti–rat IgG–coated magnetic beads (Bio-Rad). Purified T cells were labeled with 5 μM CellTrace Violet (Thermo Fisher Scientific). Enriched OT-I T cells were determined by flow cytometry to be 90–95% CD8^+^ TCRVα2^+^ cells.

Purified CD11c^+^ DCs were incubated with 2 x 10^−3^ ng/mL ovalbumin (OVA)_254-267_ peptide SIINFEKL. Pulsed DCs were titrated and co-cultured with 50 x 10^3^ CellTrace Violet-labeled OT-I T cells in complete RPMI 1640 containing 20 ng/mL GM-CSF (PeproTech). T cell proliferation was determined three days later using flow cytometry in the presence of calibration beads (Spherotec). Data were analyzed with FlowJo software (Tree Star). To assess MHC I presentation of exogenous antigen, C57BL/6 splenocytes were incubated with 10 mg/mL ovalbumin (Sigma-Aldrich) prior to titrating and co-culturing with 25 x 10^4^ purified CD11c^+^ CD8^+^ DCs and 50 x 10^3^ CellTrace Violet-labeled OT-I T cells in complete RPMI 1640 containing 20 ng/mL GM-CSF (PeproTech). T cell proliferation was determined three days later by CellTrace Violet dilution.

## Results

### Reduced MHC I surface expression in MARCH1-deficient haemopoietic cells

**Fig 1:**
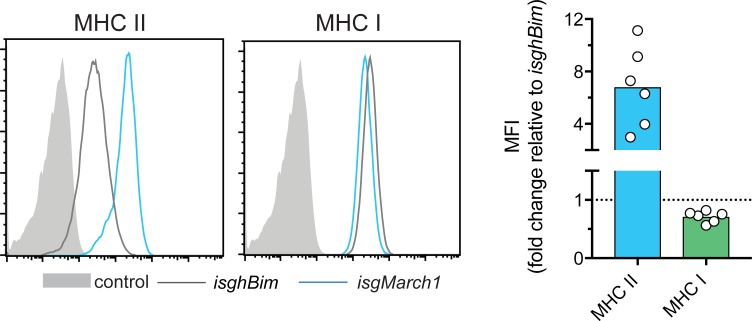
Reduced MHC I surface expression in MARCH1-deficient MuTu DC line. MHC II and MHC I expression was examined by flow cytometry for *isghBim* and *isgMarch1* MuTu DCs. The graph displays the fold-change in MFI for MHC II or MHC I expressed by *isghMarch1* cells relative to *isghBim* cells. Each symbol represents an independent experiment. MFI, mean fluorescence intensity. Control is fluorescence minus one (FMO).

To investigate the E3 ubiquitin ligase MARCH1 in antigen presentation we deleted the *March1* gene from the MuTu DC line [20] (*isgMarch1*) using CRISPR/Cas9 technology. First MuTu DCs expressing the endonuclease Cas9 were generated. These cells were then transduced to enable *March1* deletion via doxycycline-inducible small guide RNA (sgRNA). Negative control cells were generated expressing an irrelevant sgRNA targeting human (not mouse) Bim (*isghBim)*. In accordance with previous studies [8,9,11], we observed elevated surface MHC II expression in *isgMarch1* relative to *isghBim* (Fig 1). This is due to the role of MARCH1-mediated ubiquitination in enhanced turnover and reduced surface expression of this receptor. Our analysis included MHC I as a control. Surprisingly though, deletion of *March1* in MuTu DCs resulted in a small loss of MHC I from the cell surface (Fig 1). This was unexpected and prompted us to examine in further detail the influence of MARCH1 on MHC I expression using primary cells.

**Fig 2:**
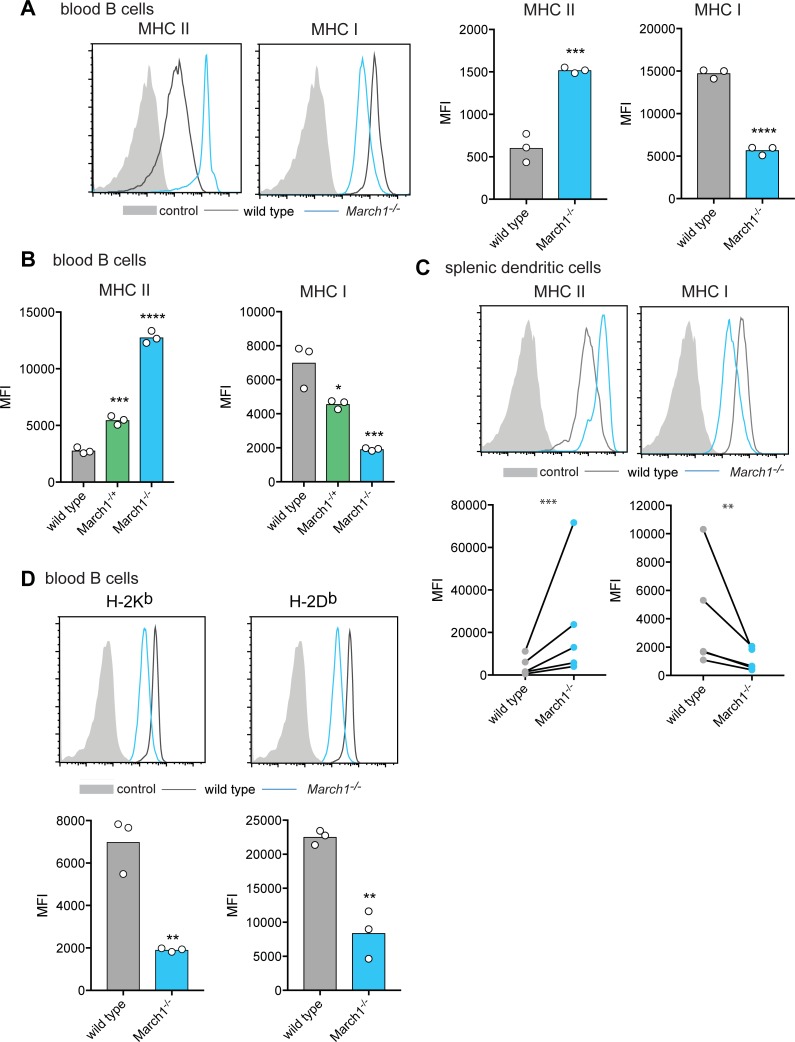
Reduced MHC I surface expression in MARCH1-deficient primary haemopoietic cells. (A) MHC II and MHC I surface expression was examined by flow cytometry for blood B220^+^ B cells. Graph displays an individual experiment where each symbol is data obtained from single mouse. Data is representative of 5 independent experiments with a total of 17 wild type mice and 13 *March1*^*-/-*^ mice. Statistical analysis was performed using an unpaired Student’s t test. (B) MHC II and MHC I surface expression was examined by flow cytometry for blood B220^+^ B cells. Graphs display an individual experiment where each symbol is data obtained from a single mouse. Data is representative of 2 independent experiments with a total of 6 *March1*^*+/+*^, 5 *March1*^*-/+*^ and 6 *March1*^*-/-*^ mice. Statistical analysis was performed using one-way ANOVA with Tukey’s multiple comparisons test. (C) MHC II and MHC I surface expression was examined by flow cytometry for splenic CD11c^+^ DCs. Data is displayed from 5 independent experiments, with each of the 5 experiments comprising 1 wild type sample (4–8 wild type spleens pooled) and 1 *March1*^*-/-*^ sample (4–8 *March1*^*-/-*^ spleens pooled). Statistical analysis was performed using a ratio paired t-test. (D) MHC I H-2K^b^ and H-2D^b^ surface expression was examined by flow cytometry for blood B220^+^ B cells. Graphs display an individual experiment where each symbol is data obtained from a single mouse. Data is representative of 3 independent experiments with a total of 9 wild type mice and 6 *March1*^*-/-*^ mice. Statistical analysis was performed using an unpaired Student’s t test (A, C and D). Control is fluorescence minus one (FMO). **P < 0.01, ***P < 0.001, ****P < 0.0001. ns, not significant. MFI, mean fluorescence intensity.

As previously shown [8,9,11], we observed elevated surface MHC II expression in primary B cells and DCs deficient in *March1* (Fig 2A–2C). We also observed a marked reduction in MHC I expression in these primary cells, more so than in *isgMarch1* MuTu DC (Fig 2A–2C). A gene dosage effect was observed so that *March1*^*+/-*^ B cells displayed intermediate MHC I levels between those in wild type and *March1*^*-/-*^ cells (Fig 2B). C57BL/6 mice co-express two MHC I molecules encoded in separate loci, H-2K^b^ and H-2D^b^, and both were similarly reduced in *March1*^*-/-*^ cells (Fig 2D). These findings were surprising because MARCH1 had not been previously implicated in regulating MHC I expression.

### MARCH1 does not alter MHC I ubiquitination or surface internalization

**Fig 3:**
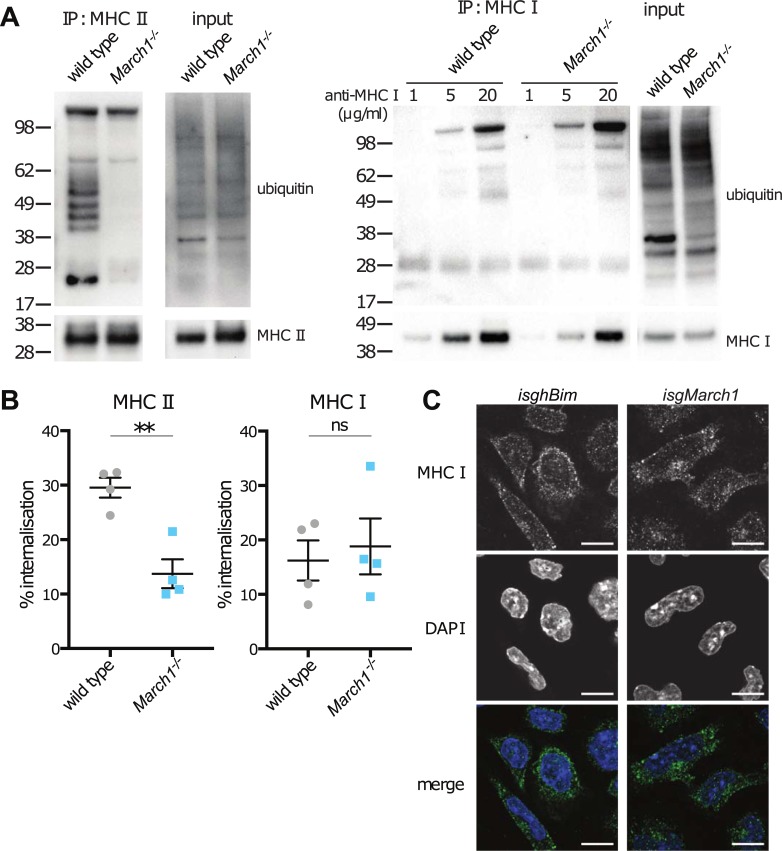
MARCH1 does not alter MHC I ubiquitination or surface internalization. (A) Wild type and *March1*^*-/-*^ spleen DCs were isolated and whole cell lysis performed (input). MHC II or MHC I was immunoprecipitated (IP) and immunoblots probed with antibodies specific for MHC II, MHC I and ubiquitin. The high MW band in the immunoblots probed for ubiquitin is non-specific. (B) Internalization of surface MHC II and MHC I was measured for wild type and *March1*^*-/-*^ spleen DCs. Cells were labeled with FIP-conjugated anti-MHC II or anti-MHC I and after 30 min of culture at 37°C, cells were exposed to quencher (Q). Percentage internalization was calculated as described in Materials and Methods. Data are pooled from 4 independent experiments performed in triplicate. Graph represents the mean internalization ± SEM. Data were analyzed using unpaired Student’s t test. **P < 0.01. ns, not significant. (C) Immunofluorescence microscopy of *isghBim* and *isgMarch1* MuTu DCs. Cells were fixed and permeabilized. Cells were stained with rabbit anti-MHC I polyclonal antiserum (generated to the cytosolic tail of MHC I) donkey anti-rabbit Alexa Fluor 647 and 0.5 μg/ml DAPI. Images were collected from 55 *isghBim* and 45 *isgMarch1* MuTu DCs.

Ubiquitination of MHC II by MARCH1 alters its rate of internalization and/or recycling leading to reduced surface expression and increased endosomal accumulation and turn-over [3,4]. Therefore, we tested the hypothesis that MARCH1 also ubiquitinated MHC I but in this case the outcome being increased, rather than decreased, plasma membrane deposition. Overall levels of MHC I were not altered in the absence of MARCH1 suggesting that the synthesis and translation of MHC I was unaffected in these cells (S2 Fig). We examined the ubiquitination status of MHC I in the presence and absence of MARCH1. MHC II served as a reference for MARCH1-dependent ubiquitination. MHC I and MHC II were immunoprecipitated from wild type or *March1*^*-/-*^ primary DCs and ubiquitinated molecules were detected by western blot. As expected, a fraction of the MHC II molecules isolated from wild type cells were covalently attached to varying units of ubiquitin (polyubiquitinated), which were revealed as a characteristic ladder of increasing molecular weight (Fig 3A). These ubiquitinated molecules were absent from MHC II immunoprecipitates obtained from *March1*^*-/-*^ DCs (Fig 3A). In contrast to MHC II, MHC I exhibited no evidence of ubiquitination in wild type or MARCH1-deficient cells, under conditions where increasing amounts of MHC I were immunoprecipitated (Fig 3A). This observation did not discard the possibility that MHC I was indeed ubiquitinated, but the modification was transient and short-lived so that the number of polyubiquitinated molecules at any given time was too small to allow detection by western blot, yet sufficient to affect MHC I trafficking and overall surface expression. Therefore, we next examined the rate of MHC I internalization from the cell surface.

We [14] and others [5,8,9,11] have previously shown that MARCH1 ubiquitination enhances the rate of MHC II internalization from the surface of B cells and DCs, via increased endocytosis, reduced recycling, or both. We hypothesized that the opposite might be true for MHC I in MARCH1-expressing cells. If this were the case, the rate of internalization of MHC I molecules from the cell surface would be expected to increase in MARCH1-deficient cells. This was investigated using DNA-based fluorescence internalization probes (FIPs) which we have previously used to monitor the fate of cell surface receptors [14,26,28]. Splenic wild type and *March1*^*-/-*^ DCs were isolated and incubated with FIP-labeled mAbs specific for MHC I or MHC II. The cells were then cultured at 37°C to enable internalization of the MHC-mAb complexes and after 30 mins the quencher-DNA probe was added to eliminate the fluorescent signal from complexes remaining on the cell surface. In this assay, the intensity of the fluorescent signal is directly proportional to the amount of internalized molecules. Rates of MHC II and MHC I internalization by wild type DCs were as in our previous analyzes [14,26]. As shown, MHC II exhibited significantly reduced internalization in *March1*^*-/-*^ DCs (Fig 3B). In contrast, the rate of MHC I internalization in wild type and *March1*^*-/-*^ DCs was comparable (Fig 3B). Furthermore, the intracellular distribution of MHC I in MuTu DCs was unaffected by the lack of MARCH1 (Fig 3C). These results indicate that MARCH1 does not ubiquitinate nor directly alter the expression or intracellular trafficking of MHC I.

### Reduced MHC I surface expression in MARCH1-deficient cells requires MHC II expression

**Fig 4:**
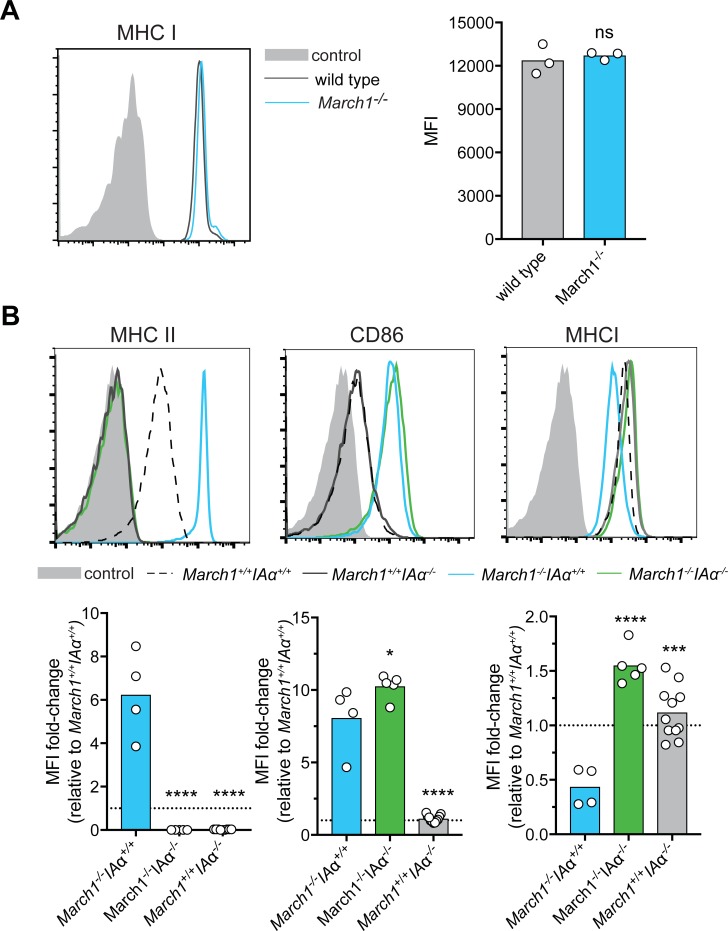
Reduced MHC I surface expression in MARCH1-deficient cells requires MHC II expression. (A) MHC I surface expression was examined by flow cytometry for blood CD4^+^ T cells. Graphs display an individual experiment where each symbol is data obtained from a single mouse. Data is representative of 4 independent experiments with a total of 12 wild type mice and 10 *March1*^-/-^ mice. Statistical analysis was performed using an unpaired Student’s t test. (B) MHC II, CD86 and MHC I surface expression was examined by flow cytometry for blood B220^+^ B cells. Data is pooled from 5 independent experiments with a total of 22 *March1*^+/+^ *I-Aα*^*+/+*^, 4 *March1*^-/-^ *I-Aα*^*+/+*^, 5 *March1*^-/-^ *I-Aα*^*-/-*^ and 11 *March1*^+/+^ *I-Aα*^*-/-*^ mice. The MFI of MHC II, CD86 and MHC I for cells isolated from *March1*^-/-^ *I-Aα*^*+/+*^, *March1*^-/-^ *I-Aα*^*-/-*^, *March1*^+/+^ *I-Aα*^*-/-*^ was expressed as a fold-change relative to the mean MFI for wild type (*March1*^+/+^ *I-Aα*^*+/+*^) cells. Statistical analysis was performed using a one-way ANOVA with Tukey’s multiple comparisons test. **P < 0.01, ***P < 0.001,**** P < 0.0001, ns, not significant. MFI, mean fluorescence intensity. (A, B) Control is fluorescence minus one (FMO).

We then examined the possibility that reduced MHC I expression in MARCH1-deficient cells was caused indirectly by increased expression of *bona fide* MARCH1 substrates, specifically MHC II. First, we examined CD4^+^ T cells isolated from *March1*^*-/-*^ that lacked the expression of MARCH1 substrate MHC II. These cells showed no significant difference in their levels of MHC I (Fig 4A). This suggested that MHC I was impacted by the aberrant trafficking of MHC II in *March1*^*-/-*^ cells. To formally address this possibility *March1*^*-/-*^ mice were intercrossed with *I-Aα*^*-/-*^ mice [23] and the surface expression of MHC II and MHC I on circulating B cells of littermates lacking expression of MARCH1, MHC II, or both, was examined by flow cytometry. In this case we included CD86 as a control, given that it is also a substrate of MARCH1. As expected, B cells from mice deficient only in MARCH1 displayed increased surface levels of MHC II and CD86, but reduced MHC I expression, compared to wild type littermates (Fig 4B). In contrast, the level of MHC I expression was restored and indeed increased in mice lacking both MARCH1 and MHC II (Fig 4B). As expected, CD86 expression was increased in *March1*^-/-^ littermates independent of MHC II expression (Fig 4B). This result indicated that the reduced level of surface MHC I level in *March1*^-/-^ cells was a consequence of increased MHC II expression.

### Reduced MHC I surface expression in MHC II K225R^ki/ki^ cells

**Fig 5:**
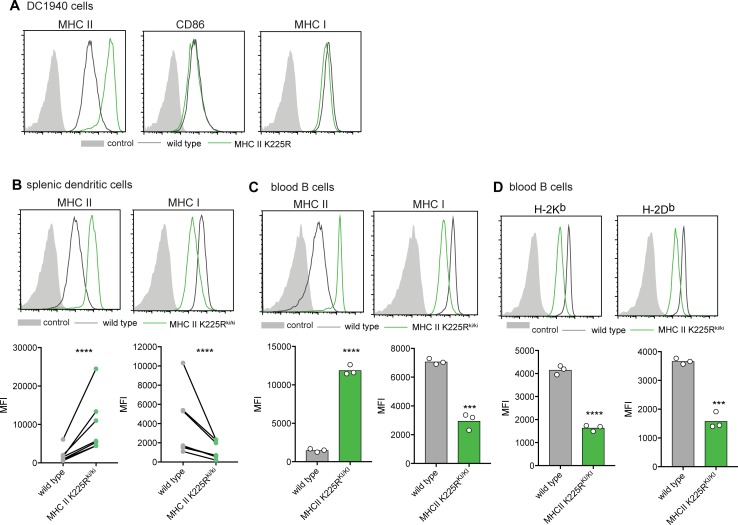
Reduced MHC I surface expression in MHC II K225R^ki/ki^ cells. (A) MHC II, CD86 and MHC I expression was examined by flow cytometry for MuTu DCs overexpressing, or not, *MHCII K225R*. Data is from 1 experiment. (B) MHC II and MHC I surface expression was examined by flow cytometry for splenic CD11c^+^ DCs. Data is displayed from 8 independent experiments, with each of the 8 experiments comprising of 1 wild type sample (4–8 wild type spleens pooled) and 1 MHC II K225R^ki/ki^ sample (4–8 MHC II K225R^ki/ki^ spleens pooled). Statistical analysis was carried out using a ratio paired t-test. (C) MHC II and MHC I expression on wild type and MHC II K225R^ki/ki^ B cells. Graphs display an individual experiment where each symbol is data obtained from a single mouse. Data is representative of 6 independent experiments with a total of 18 wild type and 18 MHC II K225R^ki/ki^ mice. (D) MHC I H-2K^b^ and H-2D^b^ expression on blood-circulating B cells. Graphs display an individual experiment where each symbol is data obtained from a single mouse. Data is representative of 3 independent experiments with a total of 9 wild type and 9 MHC II K225R^ki/ki^ mice. (B-D) Statistical analysis was performed using unpaired Student’s t tests, *** P < 0.001**** P < 0.0001. MFI, mean fluorescence intensity. (A-D) Control is fluorescence minus one (FMO).

To test whether high surface MHC II expression was sufficient to cause decreased surface MHC I expression, we examined cells isolated from mice expressing a mutant MHC II molecule that could not be ubiquitinated [18]. The Lys in position 225 of MHC II-ß is the only acceptor residue of ubiquitin in MHC II, so if this amino acid is mutated to Arg, the resulting molecule (MHC II K225R) cannot undergo ubiquitination. Cells expressing this mutant molecule display high levels of MHC II on their surface even though they still exhibit active MARCH1 as evidenced by regulation of CD86 expression [5,8,18,29]. We predicted that MHC I would be reduced in MHC II K225R cells. Indeed, MHC II K225R MuTu DCs and circulating MHC II K225R^ki/ki^ B cells and DCs possessed elevated surface MHC II, while MHC I was significantly reduced at the cell surface (Fig 5A–5C). Reduced MHC I was observed for both H-2K^b^ and H-2D^b^ (Fig 5D). Therefore, surface MHC I is significantly reduced under conditions where MHC II ubiquitination is impaired.

### Altered MHC II ubiquitination reduces the ability of dendritic cells to present antigen via MHC I

**Fig 6:**
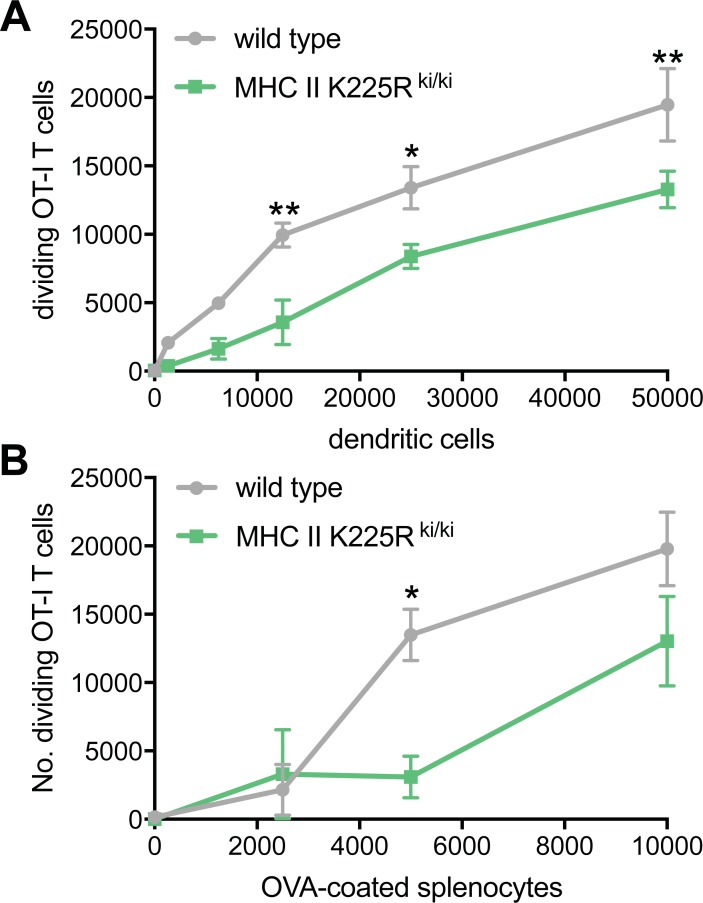
Altered MHC II ubiquitination reduces the ability of dendritic cells to present antigen via MHC I. (A) Purified wild type or MHC II K225R^ki/ki^ spleen CD11c^+^ DCs were pulsed with SIINFEKL peptide. SIINFEKL-pulsed DCs were incubated with CellTrace Violet-labeled OT-I T cells for 3 days. The number of dividing CD8^+^ TCRV*α*2^+^ OT-I T cells was determined by flow cytometry. (B) Purified spleen CD8^+^ CD11c^+^ cDC1 wild type and MHC II K225R^ki/ki^ were incubated with CellTrace Violet-labeled OT-I T cells. DCs were pulsed with OVA-coated splenocytes. The number of dividing CD8^+^ TCRV*α*2^+^ OT-I T cells was determined by flow cytometry following three days of cell culture. (A, B) Graphs display mean ± SEM. Data is representative of 2 independent experiments, performed in triplicate. *P < 0.05, **P < 0.01.

Finally, we assessed the consequences of altered MHC II ubiquitination on MHC I antigen presentation. To do this, first we tested the capacity of purified primary DCs to present peptide antigen to CD8^+^ T cells. Measuring peptide presentation eliminated potential differences caused by altered antigen processing in cells deficient in MARCH1. We tested presentation of ovalbumin (OVA)_254-267_ peptide SIINFEKL to OT-I CD8^+^ T cells that recognize H-2K^b^-SIINFEKL complexes [24]. Equal numbers of DCs purified from wild type or MHC II K225R^ki/ki^ mice were pulsed with SIINFEKL peptide. MHC II K225R^ki/ki^ DC displayed similar levels of co-stimulatory molecules compared to wild type DC (S3 Fig). Exogenous peptide will replace endogenous peptides bound to MHC I molecules at the cell surface. Peptide-pulsed DCs were incubated with CellTrace Violet-labeled OT-I CD8^+^ T cells to assess their proliferative capacity by flow cytometry. In accordance with their lower surface MHC I expression, MHC II K225R^ki/ki^ DCs pulsed with SIINFEKL peptide displayed a significantly reduced capacity to stimulate the division of OT-I T cells compared with wild type DCs (Fig 6A).

We next assessed MHC I cross-presentation of intact cell-associated antigen. In this case, the cDC1 subset of splenic DC (also known as CD8^+^ DCs) that specialize in cross-presentation was purified from wild type or MHC II K225R^ki/ki^ mice. cDC1 were incubated in the presence of cell-associated OVA protein (OVA-coated splenocytes) *in vitro* and the capacity of the different DC populations to present ovalbumin (OVA)_254-267_ peptide SIINFEKL to CellTrace Violet-labeled OT-I CD8^+^ T cells assessed by flow cytometry. MHC II K225R^ki/ki^ DCs had a reduced capacity to cross-present OVA (Fig 6B). Therefore, altering MHC II ubiquitination significantly reduces presentation of both peptide and cell-associated antigen via MHC I.

## Discussion

Here we describe an unexpected role for MHC II ubiquitination in regulating MHC I surface expression. This is a role that has not previously been reported and reveals a mechanism that coordinates the intracellular trafficking pathways involved in the MHC I and MHC II antigen presentation.

MHC I is a known substrate of viral-encoded E3 ubiquitin ligases that promote its ubiquitination and degradation. Kaposi's sarcoma-associated herpes virus and murine γ-herpes virus both encode viral homologues of the MARCH E3 ubiquitin ligases that ubiquitinate MHC I, together with other immune molecules, as a major mode of viral immune evasion [30]. MHC I is also a reported substrate of MARCH E3 ubiquitin ligases, although this was detected following E3 ubiquitin ligase overexpression systems which may yield spurious ubiquitination [31]. Analysis performed with activated DCs shows that MHC I is ubiquitinated by MARCH9 in the trans Golgi network promoting its transport to endosomal compartments [32]. Thus it was a reasonable hypothesis that MARCH1 ubiquitinated MHC I and this somehow enhanced its expression at the cell surface. In resting DCs, however, we found no evidence for MHC I ubiquitination in the presence or absence of MARCH1.

Surprisingly, the cause of lower surface MHC I in the absence of MARCH1 was directly correlated with increased surface MHC II expression in the absence of its ubiquitination. What is the mechanism involved? Currently this is unclear however there are a number of speculative possibilities. MHC II resides at the cell surface in specialized microdomains enriched in tetraspanin proteins [33] and can cluster with MHC I [34,35]. One possibility is that excess MHC II molecules disturb the microdomains such that MHC I is rendered vulnerable to destabilization. Indeed, a lack of MHC II ubiquitination has been shown to lead to MHC II “proteotoxicity” with disruption to lipid rafts and altered tetraspanin expression at the cell surface [36]. Alternatively, MHC II and MHC I may compete for the recycling pathway. When ubiquitinated, MHC II is no longer recycled to the cell surface and is instead directed to lysosomes for degradation [5,9,11,36]. An influx of non-ubiquitinated MHC II molecules into the recycling pathway may impair MHC I recruitment and increase delivery to lysosomes. However, there are conditions where high MHC II at the cell surface does not elicit low MHC I. During DC activation high MHC II is accompanied by high MHC I expression [37]. This scenario involves a shut down in MHC II synthesis accompanied by an increase in MHC I synthesis [27,37–39] and a shut down of MARCH 1 expression [9,10]. In addition there is the potential for activation signals to alter tetraspanin function [40] and/or promote re-organization of plasma membrane microdomains [41]. Altogether this may enable activated DCs to accommodate an increase in surface MHC II together with MHC I. Irrespective of the mechanism involved, MHC I may not be the only molecule affected as previous reports also describe reduced CD18 expression at the surface of *March1*^-/-^ and MHC II K225R^ki/ki^ cells [19]. Finally, MARCH ligases are regulated by CD83 [14,42] and therefore, CD83 has the potential to indirectly control MHC I surface expression.

Importantly, failure of DCs to control MHC II trafficking by ubiquitination in K225R^ki/ki^ DCs elicited a surprising reduction in MHC I cross-presentation of both peptide and cell associated antigen. This suggests that the functionally independent MHC I and MHC II antigen presentation pathways are in fact coordinated. Therefore, we have identified the MARCH1 E3 ligase as having the potential to regulate both MHC I and MHC II antigen presentation. We also report that this mechanism occurs in B cells and therefore would also expect their ability to present antigen via MHC I to be compromised, although the impact this might have on CD8^+^ T cell immunity is uncertain. In summary, our findings implicate a previously unrecognized role for MHC II ubiquitination in the regulation of CD8^+^ T cell responses. Therefore, the regulation of immunoreceptor endocytic trafficking can have unexpected consequences for immunity.

## Supporting information

S1 Fig Gating strategies for flow cytometric analyses. Cells were gated on forward- and side-scatter before doublets were excluded. Dead cells were then excluded (PI^+^). (A) MuTu DCs were further identified based on their expression of GFP, mCherry (cas9vector) and CFP (gRNA vector). (B) Blood circulating B cells were identified as B220^+^ CD3^-^ whereas splenic T cells were identified as CD3+ followed by separation of CD4^+^ and CD8^+^ T cells based on their expression of CD4 or CD8 respectively. (C) Lymph node T cells, purified for antigen presentation assays, were identified as CD8^+^ and TCRV*α*2^+^. (D) Splenic conventional dendritic cells, isolated for FACS analysis, were identified as CD11c^+^.
(EPS)

S2 Fig Whole cell lysate expression of MHC I. (A) Equal numbers of isghBim and isgMarch1 MuTu DC1940 cells were lysed with NP40 lysis buffer. Lysates were denatured and titrated dilutions were separated using SDS-PAGE. Blots were probed with anti-MHC I (polyclonal rabbit anti-serum) and anti-actin (A5060) as indicated. (B) The band intensities for MHC I in the 1:1 dilution were normalised to the intensity of the actin band in 3 independent experiments. Graph represents the mean MHC I/actin expression ± SEM. Statistical analysis was performed using an unpaired Student’s t test. ns = not signficant.
(EPS)

S3 Fig MHC and costimulatory molecules expressed by wild type and MHC II K225R^ki/ki^ CD8^+^ and CD8^-^ CD11c^+^ splenic dendritic cells. Dendritic cells were isolated from the spleen of wild type and MHC II K225R^ki/ki^ mice and analysed by flow cytometry.
(EPS)

## Abbreviations

CRISPR: clustered regularly interspaced short palindromic repeats
DC: dendritic cell
cDC1: conventional DC 1
FIP: fluorescence internalization probe
isg: inducible small guide RNA
isgh: inducible small guide human RNA
ki: knock in
MARCH1: membrane associated RING-CH 1
MFI: mean fluorescence intensity
crRNA: CRISPR targeting RNA
sgRNA: single guide RNA
tracrRNA: trans-activating CRISPR targeting RNA
